# Occupational heavy lifting and risk of ischemic heart disease and all-cause mortality

**DOI:** 10.1186/1471-2458-12-1070

**Published:** 2012-12-11

**Authors:** Christina B Petersen, Louise Eriksen, Janne S Tolstrup, Karen Søgaard, Morten Grønbæk, Andreas Holtermann

**Affiliations:** 1 National Institute of Public Health, University of Southern Denmark, Øster Farimagsgade 5A, 1353, Copenhagen, Denmark; 2 Institute of Sports Science and Clinical Biomechanics, University of Southern Denmark, Odense, Denmark; 3 National Research Centre for the Working Environment (NFA), Lersø Parkallé 105, 2100, Copenhagen, Denmark

## Abstract

**Background:**

Occupational heavy lifting is known to impose a high cardiovascular strain, but the risk of ischemic heart disease (IHD) from occupational heavy lifting is unknown. The objective was to investigate the association between occupational heavy lifting and risk of IHD and all-cause mortality, and the influence of occupational and leisure time physical activity on this association.

**Methods:**

Data were analyzed from 1987, 1994, and 2000 from the Danish National Health Interview Surveys providing a sample of 6,692 working men and 5,921 working women aged 16–85 years without cardiovascular disease at baseline. Conventional risk factors for the outcomes IHD and all-cause mortality were controlled for in Cox analyses.

**Results:**

Among men, heavy lifting was associated with increased risk for IHD (hazard ratio (HR): 1.52, 95% Confidence interval (95% CI): 1.15, 2.02), while a decreased risk was associated with occupational (HR: 0.50, 95% CI: 0.37, 0.68) and leisure time (HR: 0.73, 95% CI: 0.56, 0.95) physical activity. Referencing men with high occupational physical activity and no heavy lifting, men with high occupational physical activity and heavy lifting did not have an increased risk (HR: 1.11, 95% CI:0.68, 1.82), while men with low occupational physical activity and heavy lifting had a substantial increased risk (HR: 2.56, 95% CI:1.52, 4.32). No significant associations were found for all-cause mortality or for females.

**Conclusion:**

These findings indicate an excessive risk for IHD from occupational heavy lifting among men, particularly among those with low occupational and leisure time physical activity.

## Background

A positive association between occupational physical activity, cardiovascular disease and mortality has been found in several cohorts [1-7]. However, the specific types of occupational physical activity conferring the increased risk for cardiovascular disease remain unsettled [8]. Identification of the types of occupational physical activity imposing the increased risk for cardiovascular disease is essential for designing effective preventive initiatives.

One previous retrospective case–control study has indicated that occupational heavy lifting increases the risk for acute myocardial infarction while occupational walking and leisure time physical activity decrease the risk [9]. Heavy lifting is known to impose an acute static cardiovascular strain excessively raising the blood pressure. In contrast, occupational walking and leisure time physical activities are characterized by dynamic movements of large muscle groups potentially promoting cardiovascular fitness and health.

However, no prospective studies have investigated if occupational heavy lifting is an independent risk factor for cardiovascular disease and mortality. Moreover, the role of general occupational and leisure time physical activity for the association between occupational heavy lifting and cardiovascular disease and mortality is unknown.

Therefore, we investigated if occupational heavy lifting was associated with IHD and mortality, and if general occupational and leisure time physical activity modify these associations.

## Methods

### Study design and population

This study was based on data from the Danish National Health Interview Surveys which were carried out in 1987, 1994, and 2000 by the National Institute of Public Health, University of Southern Denmark. The surveys consist of national representative random samples of the Danish population aged 16 or above selected from the Danish Civil Registration System. Data were collected by personal interviews and self-administrated questionnaires. Response rates were 80% in 1987, 78% in 1994, and 74% in 2000 [10]. If a person participated in more than one survey, only data from the first survey was included. In total, 22,672 men and women participated in the surveys. Unemployed participants (n = 9,746) and participants with known ischemic heart disease (IHD) prior to the interview (n = 170) were excluded. Further 143 participants were excluded due to unknown vital status. Thus, the final study population consisted of 6,692 working men and 5,921 working women aged 16–85 years.

### Assessments

Data from the population-based surveys were linked to nation-wide registers by using the unique personal identification number (CPR-number). IHD incidence was based on information on both fatal and non-fatal cases. Information on cause-specific mortality was obtained from the Danish Register of Causes of Death updated until 2008-12-31 and information on disease hospitalizations was obtained from the National Patient Register updated until 2010-05-04. IHD was defined by ICD-8 codes 410–414 and ICD-10 codes I20-I25. Data on all-cause mortality were obtained from the Danish Civil Registration System updated until 2010-05-04.

Occupational heavy lifting was estimated from the question: “Are you exposed to lifting or carrying heavy burdens (minimum 10 kg) at work more than 2 days a week?” The answer options were: “yes”, “no”, or “don’t know”.

Occupational physical activity in general was estimated from the self-administrated questionnaire by the question: “Which description most precisely covers your level of physical activity at work?” Groups were defined according to the following responses: 1) Mainly sedentary work; 2) Work that require quite a bit of standing or walking activities; 3) Standing and walking most of the time with quite a bit of carrying or lifting heavy burdens; 4) Work that requires vigorous or strenuous physical activity. Due to few observations in the extremes when stratified by occupational heavy lifting, occupational physical activity level was dichotomised as follows: 1) *Low physical activity level* combining group 1 and 2 and *high physical activity level* combining group 3 and 4.

Participants were asked to state their typical level of physical activity in leisure time during the last 12 months in one of four predefined categories: 1) vigorous physical activity (strenuous activities usually involving competition or endurance training performed regularly or several times a week); 2) moderate physical activity (exercise, endurance training or heavy gardening for at least four hours a week); 3) low physical activity (walking, bicycling or other light activities for a minimum of four hours a week); 4) sedentary activities (reading, TV-watching or other sedentary activities). Level of physical activity in leisure time was dichotomised combining low and sedentary physical activity into *“low physical activity level”* and vigorous and moderate physical activity into “*high physical activity level”.* Unfortunately, many participants in the 1987-survey were not given the question regarding physical activity in leisure time and for this reason 1,946 participants were not included in the analysis of physical activity in leisure time.

Additional variables included in the analyses were age at the time of survey, education (< 10 years, 10–12 years, and >12 years), smoking (never-smoker, ex-smoker, daily smoker (1–15 cigarettes/day), and heavy smoker (>15 cigarettes/day)), alcohol consumption (<1 drinks/day, 1–2 drinks/day, 3–4 drinks/day, and >4 drinks/day), body mass index (BMI) (<25 kg/m^2^, 25–30 kg/m^2^, and > 30 kg/m^2^), hypertension (yes, no), and self-perceived stress (often, sometimes/no).

### Statistical analyses

Initial descriptive analyses involved incidence rates for IHD and all-cause mortality during follow-up and simple frequency distributions of potential confounding variables by each category of occupational heavy lifting, occupational physical activity level, and physical activity level in leisure time. The association between occupational heavy lifting, occupational physical activity and physical activity in leisure time, respectively and the incidence of IHD as well as all-cause mortality was analyzed using the Cox proportional hazards model adjusting for potential confounding factors (age, education, physical activity, smoking habits, alcohol consumption, and self-perceived stress). Further adjustment for occupational status (body mass index, self-employed, salaried worker and skilled/unskilled worker) did not alter the estimates and was therefore omitted from the analysis. Age of the participants was applied as the underlying time in the statistical model. Evaluation of the assumption about proportional hazards was done by visual inspections of log-log plots and tested using Schoenfeld residuals. A combined measure of occupational heavy lifting and general occupational physical activity as well as a combined measure of occupational heavy lifting and physical activity in leisure time were evaluated in relation to the risk of IHD and all-cause mortality. All analyses were carried out for men and women, separately. The number of participants in the different tables may vary due to differences in the number of missing observations in the separate analyses. In all tests, *P* values were 2 sided and statistical significance was defined as p < 0.05. Analyses were performed using Stata 11.0 (StataCorp LP, Texas, USA).

## Results

During the study period 538 (8.0%) men and 215 (3.6%) women were diagnosed with IHD and 521 (7.8%) men and 260 (4.4%) women died.

Table 1, 2, and 3 show selected characteristics of the study participants stratified by occupational heavy lifting, occupational physical activity, and physical activity in leisure time, respectively. Men as well as women with occupational heavy lifting were more likely to have a low educational level, have a high occupational physical activity level, smoke heavily but less likely to drink alcohol compared to those with no occupational heavy lifting (Table 1). Furthermore, women with occupational heavy lifting were more likely to be obese (BMI > 30) compared to women with no heavy lifting. Men exposed to occupational heavy lifting were less likely to be hypertensive and stressed compared to men unexposed to heavy lifting. Occupational heavy lifting was not associated to physical activity in leisure time.

**Table 1:** Demographic, social and lifestyle related factors according to occupational heavy lifting for Men (n = 6,659) and Women (n = 5,850) in the Danish National Health Interview Surveys From 1987, 1994, and 2000

**Characteristics**		**Men**			**Women**		
		**Heavy lifting (n=2,655)**	**No heavy lifting (n=4,004)**	***P***	**Heavy lifting (n=1,576)**	**No heavy lifting (n=4,274)**	***P***
**Age** [median, (SD)]		38.9 (12.5)	42.7 (11.6)	<0.01	38.8 (11.0)	40.9 (11.3)	<0.01
**Educational level** [%]							
	< 10 years	20.5	11.6	<0.01	19.2	12.6	<0.01
	10-12 years	45.8	27.6		33.6	24.3	
	13+ years	33.7	60.8		47.2	63.1	
**Occupational physical activity** [%]							
	Low	22.4	89.2	<0.01	18.4	87.5	<0.01
	High	77.6	10.8		81.7	12.5	
**Physical activity in leisure time** [%]							
	Low	68.3	67.1	0.35	81.5	81.9	0.73
	High	31.7	32.9		18.5	18.1	
**Alcohol consumption** [%]							
	<1 drinks/day	54.0	50.1	<0.01	76.0	68.9	<0.01
	1-2 drinks/day	25.5	28.3		16.0	21.4	
	3-4 drinks/day	11.0	13.0		5.3	7.0	
	>4 drinks/day	9.5	8.6		2.7	2.7	
**Smoking status** [%]							
	Daily smoking	18.2	18.9	<0.01	21.9	19.4	<0.01
	Heavy smoking	30.1	20.7		24.2	17.2	
	Never-smoking	33.9	36.6		38.3	42.6	
	Ex-smoking	17.8	23.8		15.5	20.8	
**BMI** [%]							
	<25 kg/m^2^	51.5	52.5	0.41	67.4	74.7	<0.01
	25-30 kg/m^2^	39.7	39.5		23.6	19.1	
	>30 kg/m^2^	8.8	8.0		9.0	6.1	
**Hypertension** [%]							
	Yes	5.6	8.1	<0.01	7.7	7.8	0.93
	No	94.4	91.9		92.3	92.2	
**Stress** [%]							
	Often	6.6	9.6	<0.01	10.6	10.7	0.98
	Sometimes /No	93.4	90.4		89.4	89.3	

Abbreviations: BMI, body mass index; n, number of participants.

Shown Numbers are Percentages or Median Values.

**Table 2:** Demographic, social and lifestyle related factors according to occupational physical activity level in Men (n = 6,639) and Women (n = 5,839) in the Danish National Health Interview Surveys From 1987, 1994, and 2000 (Percentages or Median Values)

**Characteristics**		**Men**			**Women**		
		**Low (n=3,794)**	**High (n=1,823)**	***P***	**Low (n=4,056)**	**High (n=896)**	***P***
**Age** [median, (SD)]		42.4 (11.5)	39.4 (12.8)	<0.01	40.9 (11.1)	39.2 (11.5)	<0.01
**Educational level** [%]							
	< 10 years	10.9	22.6	<0.01	10.7	22.8	<0.01
	10-12 years	28.0	46.7		23.3	34.9	
	13+ years	61.1	30.7		66.0	42.4	
**Physical activity in leisure time** [%]							
	Low	67.0	68.4	<0.30	81.6	82.6	0.40
	High	33.0	31.6		18.4	17.4	
**Alcohol consumption** [%]							
	<1 drinks/day	50.7	52.8	0.62	67.9	77.3	<0.01
	1-2 drinks/day	28.0	26.5		22.1	15.3	
	3-4 drinks/day	12.8	11.2		7.5	4.6	
	>4 drinks/day	8.5	9.5		2.6	2.8	
**Smoking status** [%]							
	Daily smoking	18.9	18.2	<0.01	19.5	21.8	<0.01
	Heavy smoking	21.6	29.2		17.1	23.3	
	Never-smoking	36.4	33.6		42.5	39.2	
	Ex-smoking	23.1	19.0		20.9	15.7	
**BMI** [%]							
	<25 kg/m^2^	52.5	51.1	0.29	74.7	68.0	<0.01
	25-30 kg/m^2^	39.5	39.9		19.4	22.6	
	>30 kg/m^2^	8.1	9.1		5.9	9.4	
**Hypertension** [%]							
	Yes	8.2	5.4	<0.01	7.9	7.8	0.84
	No	91.8	94.6		92.1	92.2	
**Stress** [%]							
	Often	9.9	5.9	<0.01	11.2	9.5	<0.01
	Sometimes /No	90.2	94.1		88.8	90.5	

Abbreviations: BMI, body mass index; n, number of participants.

**Table 3:** Demographic, social and lifestyle related factors according to physical activity in leisure time in Men (n = 5,660) and Women (n = 5,029) in the Danish National Health Interview Surveys From 1987, 1994, and 2000 (Percentages or Median Values)

**Characteristics**		**Men**			**Women**		
		**Low (n=3,822)**	**High (n=1,838)**	***P***	**Low (n=4,120)**	**High (n=909)**	***P***
**Subjects** [*n*, (%)]							
**Age** [median, (SD)]		43.0 (12.0)	38.4 (11.5)	<0.01	41.1 (11.5)	38.0 (11.3)	<0.01
**Educational level** [%]							
	< 10 years	16.7	8.8	<0.01	13.1	18.2	<0.01
	10-12 years	36.0	31.8		26.7	32.8	
	13+ years	47.3	59.4		60.2	49.1	
**Alcohol consumption** [%]							
	<1 drinks/day	50.4	55.3	<0.01	70.3	71.7	0.51
	1-2 drinks/day	27.1	26.8		20.2	20.4	
	3-4 drinks/day	12.9	10.3		6.8	5.7	
	>4 drinks/day	9.6	7.6		2.8	2.2	
**Smoking status** [%]							
	Daily smoking	18.0	16.4	<0.01	19.2	18.2	<0.01
	Heavy smoking	27.0	19.8		19.9	11.9	
	Never-smoking	32.9	42.8		41.0	48.7	
	Ex-smoking	22.2	21.0		19.9	21.3	
**BMI** [%]							
	<25 kg/m^2^	46.9	57.8	<0.01	69.3	77.0	<0.01
	25-30 kg/m^2^	42.8	36.1		22.6	17.8	
	>30 kg/m^2^	10.4	6.1		8.1	5.2	
**Hypertension** [%]							
	Yes	8.2	5.4	<0.01	8.9	4.4	<0.01
	No	91.8	94.6		91.1	95.6	
**Stress** [%]							
	Often	9.0	8.0	0.20	11.4	9.4	0.08
	Sometimes /No	91.0	92.0		88.6	90.6	

Abbreviations: BMI, body mass index; n, number of participants.

Overall, the association between occupational physical activity and selected socio-demographic and lifestyle related factors were similar to the associations for occupational heavy lifting (Table 2).

Study participants, men as well as women, with a high compared to a low level of physical activity in leisure time were more likely to have a high educational level, be never-smokers, to have a BMI below 25 kg/m^2^, and to have a normal blood pressure (Table 3). Additionally, men with a high compared to low physical activity level in leisure time were more likely to drink less alcohol.

The associations between various forms of physical activity and IHD incidence as well as all-cause mortality are shown in Table 4. Men exposed to occupational heavy lifting had a higher risk of IHD compared to those who were unexposed (HR = 1.52, 95% CI: 1.15, 2.02). No associations between occupational heavy lifting and either IHD or all-cause mortality were found in women.

**Table 4:** Rates and hazard ratios (HR) of ischemic heart disease (IHD) and all-cause mortality by heavy lifting activities, occupational physical activity level, and physical activity level in leisure time at work for men and women in the Danish National Health Interview Surveys From 1987, 1994, and 2000

**Physical activity**		**IHD**						**All-cause mortality**					
		**No of cases**	**Rate**^**a**^	**HR**^**b**^	**95% CI**	**HR**^**c**^	**95% CI**	**No of cases**	**Rate**^**a**^	**HR**^**b**^	**95% CI**	**HR**^**c**^	**95% CI**
**Men**													
Heavy lifting (n=6,659)													
	No	343	6.3	1.00	Reference	1.00	Reference	179	6.3	1.00	Reference	1.00	Reference
	Yes	193	5.4	1.09	0.91, 1.30	1.52	1.15, 2.02	341	5.1	1.00	0.84, 1.20	1.00	0.74, 1.35
Occupational physical activity (n= 6,639)													
	Low	369	6.7	1.00	Reference	1.00	Reference	317	5.7	1.00	Reference	1.00	Reference
	High	162	4.7	0.82	0.68, 0.99	0.50	0.37, 0.68	201	5.9	1.13	0.94, 1.35	1.02	0.75, 1.39
Physical activity in leisure time (n=5,660)													
	Low	73	6.3	1.00	Reference	1.00	Reference	269	5.9	1.00	Reference	1.00	Reference
	High	292	3.2	0.70	0.54, 0.91	0.73	0.56, 0.95	58	2.6	0.69	0.52, 0.92	0.72	0.54, 0.96
**Women**													
Heavy lifting (n=5,850)													
No		158	6.3	1.00	Reference	1.00	Reference	197	6.3	1.00	Reference	1.00	Reference
Yes		56	5.4	1.15	0.84, 1.56	0.81	0.50, 1.56	63	5.1	1.11	0.83, 1.48	0.95	0.59, 1.53
Occupational physical activity (n= 5,839)													
Low		138	2.5	1.00	Reference	1.00	Reference	183	3.3	1.00	Reference	1.00	Reference
High		77	3.1	1.36	1.03, 1.80	1.55	0.98, 2.44	77	3.1	1.03	0-79, 1.35	0.94	0.60, 1.50
Physical activity in leisure time (n=5,029)													
Low		128	2.5	1.00	Reference	1.00	Reference	149	3.0	1.00	Reference	1.00	Reference
High		20	1.8	0.91	0.57, 1.47	0.98	0.61, 1.58	17	1.5	0.70	0.42, 1.15	0.85	0.51, 1.41

Abbreviations: CI, confidence interval; HR, hazard ratio; n, number of participants.

^a^ Pr. 1,000 person-years.

^b^ Adjusted for age.

^c^ Adjusted for age, education, alcohol consumption, smoking status, stress, heavy lifting, occupational physical activity, and physical activity in leisure time.

Men reporting a high compared to a low occupational physical activity level had a reduced risk of IHD even after adjusting for socio-demographic and lifestyle related factors including physical activity in leisure time and occupational heavy lifting (HR = 0.50, 95% CI: 0.37, 0.68) (Table 4). Among women, a high occupational physical activity level was not associated with risk for IHD compared to women with a low occupational physical activity level (HR = 1.55, 95% CI: 0.98, 2.44). No association between occupational physical activity and all-cause mortality was observed for either men or women. Physical activity level in leisure time was associated to the risk of IHD and all-cause mortality among men but not among women. Highly physically active men had a 23% reduced risk of IHD and a 26% reduced risk of all-cause mortality (HR = 0.73, 95% CI: 0.56, 0.95 and HR = 0.72, 95% CI: 0.54, 0.96). Additional analysis, showed that among men with low levels of both physical activity in occupation and in leisure time, the risk of IHD was significantly increased compared to high levels of both occupational and leisure-time physical activity (HR = 3.04, 95% CI: 1.77-5.23).

Table 5 shows the a combined measure of occupational heavy lifting and occupational physical activity level in relation to IHD and all-cause mortality. Compared to men with high level of occupational physical activity level and no lifting activities, those with low occupational physical activity who were exposed to occupational heavy lifting had a higher risk of IHD (HR = 2.56, 95% CI: 1.52, 4.32). In contrast, among men with a high level of occupational physical activity level exposure to occupational heavy lifting made no difference in the risk of IHD. In women, there seemed to be no interplay between occupational physical activity and occupational heavy lifting.

**Table 5:** Hazard ratios (HR) of ischemic heart disease (IHD) and all-cause mortality by heavy lifting activities at work stratified by occupational physical activity and physical activity in leisure time in the Danish National Health Interview Surveys From 1987, 1994, and 2000

**Occupational physical activity**	**Heavy lifting**		**IHD**					**All-cause mortality**			
		**No of cases**	**HR**^**a**^	**95% CI**	**HR**^**b**^	**95% CI**	**No of cases**	**HR**^**a**^	**95% CI**	**HR**^**b**^	**95% CI**
**Men**											
High	No	33	1.00	Reference	1.00	Reference	59	1.00	Reference	1.00	Reference
	Yes	129	1.19	0.81, 1.74	1.11	0.68, 1.82	141	0.80	0.59, 1.08	0.91	0.61, 1.36
Low	No	308	1.32	0.92, 1.89	1.49	0.93, 2.39	281	0.76	0.57, 1.00	0.90	0.61, 1.34
	Yes	60	2.05	1.34, 3.14	2.56	1.52, 4.32	36	0.75	0.50, 1.14	1.00	0.59, 1.68
**Women**											
High	No	28	1.00	Reference	1.00	Reference	39	1.00	Reference	1.00	Reference
	Yes	48	0.88	0.55, 1.40	0.78	0.45, 1.36	18	1.12	0.69, 1.80	0.92	0.50, 1.59
Low	No	130	0.69	0.46, 1.03	0.63	0.38, 1.04	166	1.03	0.68, 1.55	1.03	0.60, 1.78
	Yes	8	0.65	0.29, 1.42	0.57	0.21, 1.52	103	1.23	0.62, 2.45	1.03	0.42, 2.52
**Physical activity in leisure time**	**Heavy lifting**		**IHD**					**All-cause mortality**			
			**HR**^**a**^	**95% CI**	**HR**^**c**^	**95% CI**		**HR**^**a**^	**95% CI**	**HR**^**c**^	**95% CI**
**Men**											
High	No	50	1.00	Reference	1.00	Reference	26	1.00	Reference	1.00	Reference
	Yes	23	0.97	0.59, 1.58	1.38	0.82, 2.35	51	0.91	0.52, 1.59	0.82	0.45, 1.50
Low	No	182	1.36	1.00, 1.78	1.31	0.95, 1.81	171	1.34	0.95, 1.91	1.28	0.90, 1.83
	Yes	110	1.48	1.06, 2.07	2.05	1.39, 3.03	12	1.55	1.07, 2.25	1.33	0.87, 2.04
**Women**											
High	No	18	1.00	Reference	1.00	Reference	11	1.00	Reference	1.00	Reference
	Yes	2	0.35	0.08, 1.50	0.24	0.05, 1.06	6	1.82	0.67, 4.92	1.76	0.62, 4.98
Low	No	90	0.84	0.51, 1.41	0.79	0.47, 1.32	116	1.72	0.93, 3.20	1.44	0.77, 2.69
	Yes	38	1.17	0.67, 2.04	0.74	0.39, 1.41	33	1.65	0.83, 3.27	1.25	0.59, 2.67

Abbreviations: CI, confidence interval; HR, hazard ratio; n, number of participants.

^a^ Adjusted for age.

^b^ Adjusted for age, physical activity in leisure time, education, alcohol consumption, smoking status, and stress.

^c^ Adjusted for age, occupational physical activity, education, alcohol consumption, smoking status, and stress

Physical activity in leisure time also seemed to modify the association between occupational heavy lifting and the risk of IHD among men (Table 5). Occupational heavy lifting in combination with low physical activity in leisure time was associated with increased risk of IHD compared to men with a high leisure-time physical activity level and no occupational heavy lifting (HR = 1.97, 95% CI: 1.33, 2.91). In contrast, among men who were highly physically active in leisure-time, exposure to heavy lifting did not change the risk of IHD (HR = 1.37, 95% CI: 0.81, 2.33).

Men exposed to heavy lifting in combination with both low levels of leisure-time physical activity and occupational physical activity had almost three times higher risk of IHD (HR = 2.92) compared to men with no heavy lifting in combination with high levels of occupational and leisure-time physical activity (p = 0.04). No significant associations were seen among women or for all-cause mortality (data not shown).

## Discussion

The main finding of this study was that occupational heavy lifting was independently associated with the risk of IHD among male workers. Moreover, the risk of IHD from occupational heavy lifting was modified by both occupational and leisure time physical activity.

Male workers reporting lifting or carrying of heavy burdens of at least 10 Kg at work more than 2 days a week had 52% higher risk for IHD compared to males workers not reporting lifting or carrying of heavy burdens at work after adjusting for demographic, social and lifestyle-related factors as well as occupational and leisure time physical activity. This finding indicates that occupational lifting of heavy burdens is an independent risk factor for IHD among males. We are not aware of previous prospective cohort studies showing an independent risk for cardiovascular disease from occupational heavy lifting. However, our finding is in accordance with a previous retrospective case–control study of Fransson and colleagues, finding a tendency for an increased risk for acute myocardial infarction from occupational heavy lifting [9]. Occupational heavy lifting is well known to impose an acute cardiovascular strain excessively raising blood pressure [11]. Therefore, it is a plausible biological causation that the cardiovascular strain from heavy lifting more than two days per week over several years increases the risk for IHD.

Moreover, the association between occupational heavy lifting and IHD was shown to be modified by both occupational and leisure time physical activity among men. Men with high occupational physical activity and not performing heavy lifting did not have an increased risk for IHD, while men with low occupational physical activity and performing heavy lifting had a more than doubled risk for IHD, referencing men with high occupational physical activity not performing occupational heavy lifting. The same tendency was found for leisure time physical activity among men. Men who had a low occupational physical activity level and performed heavy lifting had an almost doubled risk of IHD, while men who had a high occupational physical activity level and also performed heavy lifting did not have an increased risk of IHD, referencing men with high occupational physical activity not performing heavy lifting. These findings indicate that occupational heavy lifting is a particularly strong hazard for IHD among men who are not being generally physically active at work or in leisure time. The positive effect of both occupational and leisure time physical activity in this study is supported by the 50% reduced risk for IHD among men with high versus low occupational physical activity, and the 27% reduced risk among men with high versus low leisure time physical activity. The observation of a modifying effect of leisure time physical activity is in agreement with our previous observation that physical activity in leisure time and physical fitness counteracts the adverse effects of occupational physical activity (including heavy lifting) on health [6,12]. However, these studies did not investigate occupational heavy lifting separately from occupational physical activity. Findings from the present study indicate that the type of activity (isometric vs. dynamic movements) is an important aspect that may determine the beneficial or adverse effects of occupational physical activity. The positive cardiovascular effects of occupational walking and leisure time physical activity are previously well documented [13-15], and can be explained by the dynamic movements of large muscle groups promoting cardiovascular fitness and health. Therefore, it is biologically plausible that high occupational and leisure time physical activity offers a protective effect against the cardiovascular strain imposed by occupational heavy lifting.

Among men, no association between occupational heavy lifting and all-cause mortality was found. Because IHD only constitutes about 25% of all deaths among men in Denmark, and that the biological causation is mediated by the negative effects of occupational heavy lifting on the cardiovascular system, we consider this finding to support the independent relation between heavy lifting and IHD among men.

Although not statistically significant, the risk of IHD by heavy lifting among women showed opposite associations than among men. Most likely, the sex differences can be explained by different work tasks for males and females imposing different physiological impacts. Factors such as work load and frequency of lifted burdens may play an important role. Unfortunately, detailed information on duration of occupational lifting as well as exact weight and frequency of the lifted burdens was not available.

### Strengths and limitations

The main strengths of this study are the population based prospective design linked to register based information and the high participation rates. The design allows adjusting for effects of several potential confounding factors. Some factors, such as BMI or stress, may be regarded as intermediate factors rather than confounding factors and adjusting for these may have conservatively biased our results. Conversely, factors such as hypertension or diabetes were not included as confounding factors as these were regarded as intermediate variables. Unmeasured factors may also influence the association, including dietary habits, which were not possible to adjust for.

A limitation of this study is the crude assessment of occupational and leisure-time physical activity by questionnaire in which participants were forced into four predefined categories possibly leading to an underestimation of the results [16,17]. Moreover, the dichotomization of physical activity may have diluted the results as it is not possible to distinguish the effect of subcategories of high and low activity levels. However, we conducted sensitivity analyses applying the four-category measure of physical activity level, and found similar although attenuated results. Finally, results should be interpreted given the limitations of self-reporting such as social desirability bias which is likely to have affected the reporting of physical activity in leisure time. However, previous studies have shown self-reported physical activity to be a fairly accurate measure when used in large epidemiological studies [16,18,19].

## Conclusions

The results of this study indicate that occupational heavy lifting is an independent risk factor for IHD among men, while this was not shown among women. Occupational heavy lifting seems to be a particularly strong risk factor among men with low occupational and leisure time physical activity. Preventive initiatives for IHD should be targeting men with occupational heavy lifting.

## Competing interests

The authors declare that they have no competing interests.

## Authors' contributions

All authors contributed to the conception, design, interpretation of data, and writing or critically revising the manuscript. CBP made the statistical analyses. CBP and AH are guarantors. All authors read and approved the final manuscript.

## Pre-publication history

The pre-publication history for this paper can be accessed here:

http://www.biomedcentral.com/1471-2458/12/1070/prepub
